# Supplementary material for: Antioxidant properties of dihydroxy B-ring flavonoids modulate circadian amplitude in Arabidopsis
Source: Life Sci Alliance. 2025 Sep 29;8(12):e202503328. doi: 10.26508/lsa.202503328 (PMC12479380; doi:10.26508/lsa.202503328)
Supplement: Supplementary file 3 [file LSA-2025-03328_TableS1.docx]

**Table S1. Primers used for genotypic confirmation of Col-0 and *tt4-2* aequorin reporter lines.**

| **Primer Name** | **Sequence (5’ to 3’)** |
| --- | --- |
| Aequorin MAQ2 Forward | ATGACCAGCGAACAATACTCAGT |
| Aequorin MAQ6 Forward | GCTTTGCAATTCATACAGAAGTGAG |
| Aequorin Reverse | TTAGGGGACAGCTCCACCGTA |
